# Continuous Improvement of Frontal Rhytids Following Glabella Only Treatments With Neuromodulators—A Clinical Prospective Pilot Study

**DOI:** 10.1111/jocd.70188

**Published:** 2025-08-07

**Authors:** Victor R. M. Munoz‐Lora, Victor Rogerio, Vanessa Thiesen, Ana C. N. Carnevali, Carlos Bravo, Sachin M. Shridharani, David Eccleston, Michael Alfertshofer, Marcelo Germani, Sebastian Cotofana

**Affiliations:** ^1^ Department of Periodontology and Implantology University of Guarulhos São Paulo Brazil; ^2^ Let's HOF Academy São Paulo Brazil; ^3^ Private Practice Sao Paulo Brazil; ^4^ Private Practice San Jose Costa Rica; ^5^ Division of Plastic and Reconstructive Surgery Washington University St. Luis Missouri USA; ^6^ Private Practice New York New York USA; ^7^ Private Practice Birmingham UK; ^8^ Department of Oral and Maxillofacial Surgery Charité ‐ Universitätsmedizin Berlin Berlin Germany; ^9^ Department of Biological Sciences, Bauru School of Dentistry University of São Paulo Sao Paulo Brazil; ^10^ Department of Plastic Surgery Vanderbilt University Medical Center Nashville Tennessee USA; ^11^ Department of Dermatology Erasmus University Medical Center Rotterdam the Netherlands; ^12^ Centre for Cutaneous Research Blizard Institute, Queen Mary University of London London UK; ^13^ Department of Plastic and Reconstructive Surgery Guangdong Second Provincial General Hospital Guangzhou Guangdong China

**Keywords:** facial aesthetics, facial biomechanics, forehead lines, glabellar lines, neuromodulator treatments

## Abstract

**Introduction:**

While traditionally focused on treating glabellar and forehead rhytids through direct neuromodulator injections, recent findings on the biomechanics of facial muscles suggest that glabellar treatments alone may influence forehead wrinkles by altering the dynamic balance between depressor and elevator muscles.

**Objective:**

To evaluate whether glabellar‐only neuromodulator treatments can reduce forehead wrinkle severity without direct injections into the frontalis muscle.

**Methods:**

This prospective, interventional study included 18 participants with moderate to very severe glabellar lines. Neuromodulator (AbobotulinumtoxinA; 37.5 sU (= 15 IU)) injections were administered exclusively in the glabella at three intervals over 7 months. Glabellar Severity Scale (GLSS), Forehead Wrinkle Scale (FWS), Frontal Skin Displacement (FSD), and Eyebrow Position Scoring (EPS) were assessed at baseline and 30 days after each cycle.

**Results:**

GLSS scores significantly improved across treatment cycles (baseline: 3.0; post‐third cycle: 1.0; *p* < 0.001). FWS improved from 3.0 at baseline to 1.0 after the third cycle (*p* = 0.005), while FSD showed a significant reduction from 37.2 mm to 17.9 mm (*p* < 0.01). No changes in eyebrow position were detected following EPS assessment.

**Conclusion:**

Glabellar‐only neuromodulator treatments with 37.5 sU (= 15 IU) effectively reduced forehead rhytid severity, likely by altering the balance between depressor and elevator muscles. This approach minimizes risks associated with direct frontalis injections and offers a novel strategy for forehead rejuvenation. Moreover, the observed progressive improvement across treatment cycles suggests that this strategy may continuously enhance aesthetic outcomes over time, supporting the rationale for maintenance treatments at regular intervals.

## 1 Introduction

According to the annual statistic released by the American Society of Plastic Surgeons, botulinum toxin injections remain the most frequently performed non‐surgical aesthetic procedure in the US, with 9 480 949 treatments administered in 2023. These numbers underscore the growing popularity of neuromodulators in facial aesthetics and their widespread use for addressing facial rhytids [1].

Neuromodulators have been extensively used in the upper third of the face to treat static and dynamic wrinkles, particularly of the glabella and forehead [2]. The classic treatment algorithm involves direct injections into the frontalis muscle to reduce horizontal forehead lines [3] and into the glabellar complex to smooth vertical and horizontal lines caused by contractions of procerus, corrugator supercilii, and orbicularis oculi muscles.

In 2022, Cotofana et al. [4] introduced the concept of the “axes of movement” relevant for eyebrow positioning by describing pairs of eyebrow elevators and eyebrow depressors which form a system of agonistic and antagonistic muscles to precisely positing eyebrows during various facial expressions. This theory was confirmed in 2024 by Rams and his colleagues in an elegant magnet resonance imaging study which found out that during facial expressions eyebrow elevators and eyebrow depressors contract at the same time simultaneously and not only when their specific activity is required [5]. Their finding is novel because until now it was assumed that when elevating the eyebrow only frontalis muscle was active and not its respective antagonists.

Translating their finding into clinical practice, it can be assumed that when activating eyebrow depressors during the facial expressions of frowning or looking angry (= activation of procerus, corrugator supercilii, and orbicularis oculi muscles) the respective elevator (= frontalis muscle) is active as well. In consequence, it could be assumed that when treating the glabella with neuromodulators (= relaxation of the depressors) the corresponding elevator muscle (= frontalis muscle) can contract to a lesser extent than usual to maintain the eyebrows in a desired position. This could potentially result in less horizontal forehead lines, which are a direct indicator of frontalis muscle contraction and activity [6].

To continue this line of research, the present study was designed: it is hypothesized that treatments of the glabella with neuromodulators can reduce the severity of horizontal forehead lines even if the forehead is not targeted with neuromodulators during that treatment cycle. Further, it could be assumed that multiple treatment cycles with glabella‐only treatments could result in continuous improvement of forehead wrinkle severity due to the continuous reduction in antagonistic depressor activity. To test the above hypotheses, this clinical interventional study was conducted in which the glabella only was treated with neuromodulators (and not the frontalis muscle) with a total study duration time of 7 months and three treatment cycles.

## 2 Methods

### 2.1 Study Design

This pilot prospective, interventional, non‐comparative investigation was conducted between January and November 2024. Ethical approval was obtained from the Research Ethics Committee of the Centro Universitário Católico Salesiano Auxilium–UniSALESIANO/SP (protocol number CAAE–70079723.1.0000.5379). All participants provided written informed consent prior to their inclusion in this study, and all treatments followed the guidelines of Good Clinical Practice and the standard of care according to national regulations.

**FIGURE 1:**
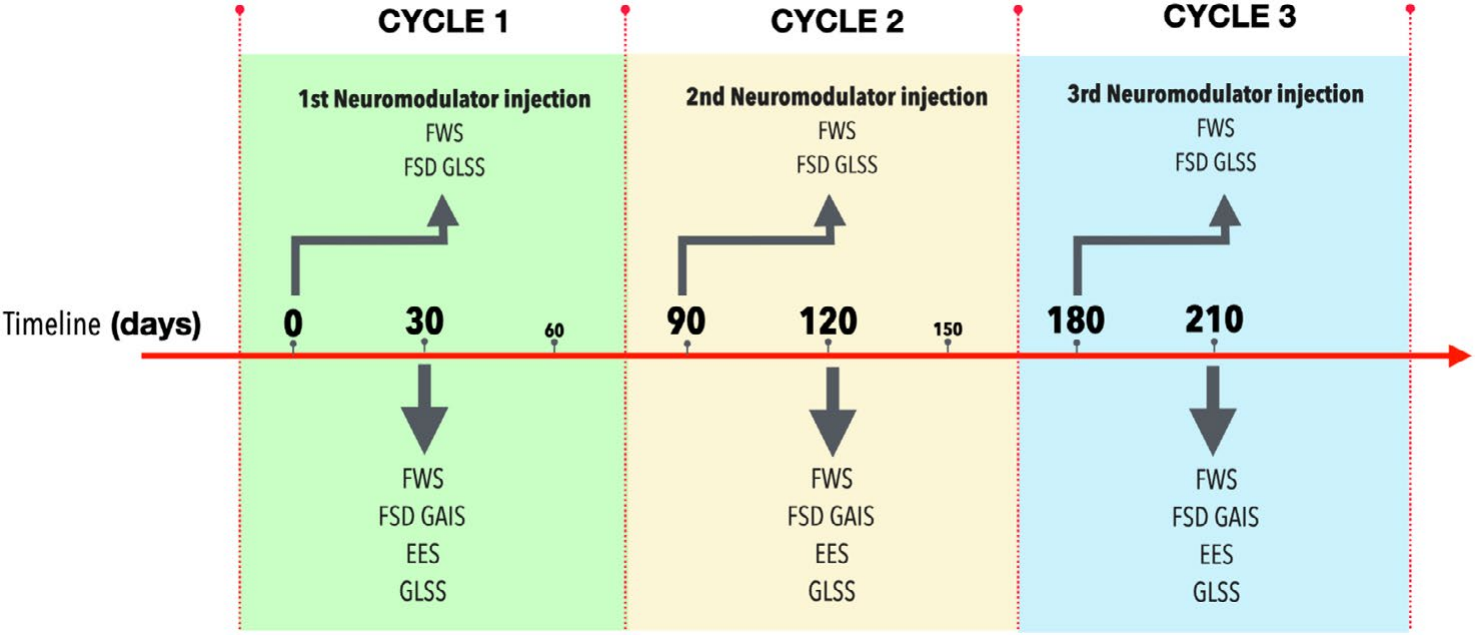
Timeline and structure of the study showing the treatment and evaluation cycles. Neuromodulator injections were administered in the glabellar region on Day 0, 90, and 180. Post‐treatment evaluations were conducted 30 days after each cycle on days 30, 120, and 210, respectively. EES, Eyebrow Elevation Scale; FSD, frontal skin displacement; FWS, Frontal Wrinkle Scale; GAIS, Global Aesthetic Improvement Scale; GLSS, Glabellar Severity Scale.

Neuromodulator treatments were exclusively administered in the glabella at three time points: Day 0, 90, and 180, completing three treatment cycles. No touch‐ups or additional interventions were performed during the study period. Post‐treatment evaluations were conducted 30 days after each treatment cycle (on Day 30, 120, and 210) to assess both objective and subjective outcomes (Figure 1). No treatment of the forehead occurred during the study observational period.

### 2.2 Study Sample

A total of 18 Brazilian, multi‐ethnic, consecutive volunteers were included in this study with moderate to very severe glabellar lines as assessed during maximal frowning according to previously published classification [7]. Patients requested a treatment for their glabellar lines and were injected following a precise 3‐point injection technique for the procerus and corrugator muscles [8]. No specific inclusion criteria were applied, allowing for a broad representation of healthy adult volunteers.

Exclusion criteria comprised individuals with pre‐existing medical conditions such as neuromuscular disorders, blood clotting issues, known allergies to neuromodulators or their components, active infections in the treatment area, and those who were pregnant or lactating. Additionally, patients who had undergone facial aesthetic surgeries or received any aesthetic treatments, including neuromodulators, within the 12 months preceding the study were not included.

### 2.3 Glabellar Injection Technique

**FIGURE 2:**
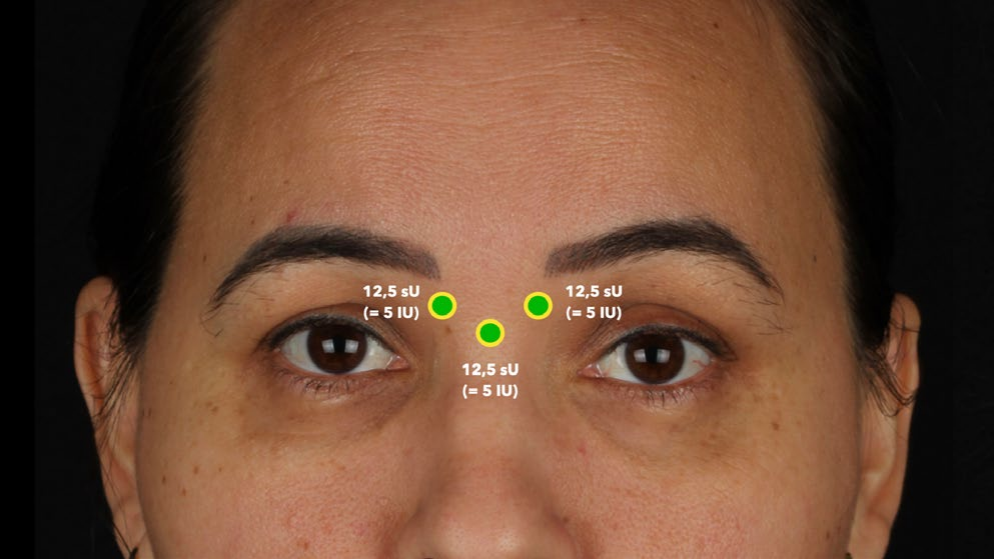
Representation of the conducted 3‐point injection technique used for glabellar treatment. sU, Speywood units.

All treatments were performed by the same experienced clinician (M.G.) with over 5 years of experience in facial toxin treatments as described previously [9, 10]. The neuromodulator used was AbobotulinumtoxinA (Dysport, Galderma, Uppsala, Sweden) that was always prepared on the day of treatment by reconstituting 500 sU (= 200 IU) of the toxin with 2.0 cc of sterile saline solution (Samtec, Ribeirão Preto, Brazil), corresponding to a concentration of 250 sU/cc. The glabellar area was cleaned with 2% chlorhexidine, and topical anesthesia (lidocaine 4%, Dermomax, Aché Laboratorios Farmacêuticos, Guarulhos, Brazil) was applied to the designated injection sites. A 31G‐6mm syringe (Becton Dickinson, Franklin Lakes, NJ, USA) was used to conduct the 3‐point injection technique as described previously by Cotofana et al. [8]. In brief, the bony origins of the procerus and of both corrugator supercilii muscles were targeted with each 12.5 sU (= 5 IU) resulting in a total glabella dose per patient of 37.5 sU (= 15 IU) of AbobotulinumtoxinA. (Figure 2).

The injections were exclusively administered in the glabella at three time points: Day 0, 90, and 180, completing three treatment cycles. No touch‐ups of the glabella or treatments of the forehead of any kind were performed during the study period.

### 2.4 Outcome Assessments

#### 2.4.1 Glabellar Severity Scale (GLSS)

Severity of glabellar rhytids was evaluated before (Day 0, 90, and 180) and 30 days after (Day 30, 120, and 210) each injection cycle using the glabellar severity scale (GLSS) using a 0 to 4 scale, where 0 = “no lines,” 1 = “mild lines,” 2 = “moderate lines,” 3 = “severe lines,” and 4 = “very severe lines” [7].

##### 2.4.1.1 Forehead Wrinkle Scale (FWS)

The severity of horizontal forehead lines during maximum contraction of the frontalis muscle was assessed before (Day 0, 90, and 180) and 30 days after (Day 30, 120, and 210) each injection cycle using the Forehead Wrinkle Scale (FWS) [7]. Patients were instructed to maximally contract their frontalis muscle, and an independent, experienced clinician rated the severity of the wrinkles on a scale from 0 to 4, where 0 = “no lines,” 1 = “mild lines,” 2 = “moderate lines,” 3 = “severe lines,” and 4 = “very severe lines” [11].

##### 2.4.1.2 Frontal Skin Displacement (FSD)

**FIGURE 3:**
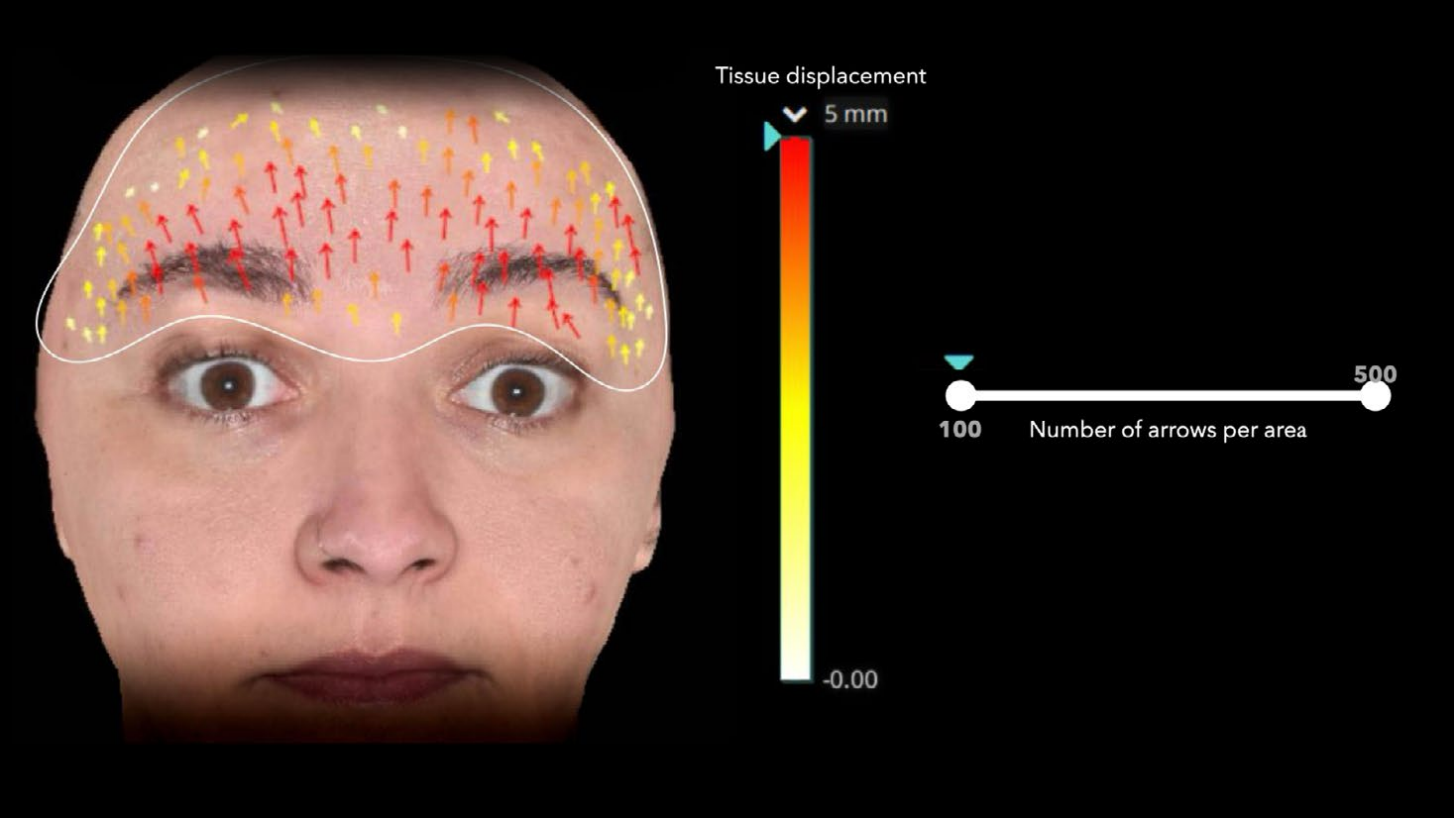
Frontal Skin Displacement Assessment Using 3D Imaging. Arrows represent the direction and magnitude of skin displacement, with reddish arrows indicating higher displacement values on a scale ranging from 0 to 5 mm.

Skin displacement of the forehead in cranio‐caudal directions was measured before (Day 0, 90, and 180) and 30 days after (Day 30, 120, and 210) each injection cycle using the 3D‐SQ device (Quantificare, Sophia Antipolis, France) as described previously [10, 12]. During the assessment, patients were instructed to perform maximum eyebrow elevation (maximum frontalis muscle contraction) while multiple stereophotogrammetric images were captured. The DermaPix Database software (Quantificare, Sophia Antipolis, France) was used to process these images and calculate variations in skin position, providing visual representations of tissue displacement via arrows. Displacement was color‐coded, with red arrows indicating the highest displacement (5 mm or more). For consistency, the analysis was standardized to 100 arrows per area, and an independent operator manually counted the arrows (Figure 3).

#### 2.4.2 Eyebrow Position Scoring (EPS)

Eyebrow position scoring (EPS) was performed to confirm the absence of neuromodulator diffusion or spread from the glabella to the frontalis muscle, ensuring that treatment effects remained localized to the eyebrow depressors and not to the eyebrow elevator (= frontalis muscle). Patients were photographed during maximal eyebrow elevation (maximum frontalis contraction) and EPS was evaluated at 30 days after each injection cycle (Day 30, 120, and 210). An independent evaluator compared these images directly to their respective baseline images in a pairwise reading: 0 vs. 30 days; 90 vs. 120 days; 180 vs. 210 days. The scoring assessed the change in eyebrow position during maximal forehead contraction using: “0” = no change, “1” = higher position than pre‐treatment, and “−1” = lower position than pre‐treatment.

### 2.5 Statistical Analysis

All statistical tests were performed according to the data type and distribution. Quantitative measurements, such as FSD, are presented as mean ± standard deviation (mean & SD), while ordinal measurements, including GLSS, FWS, and EPS, are presented as median and interquartile range (median & IQR) to align with their ordinal or nominal data format. Comparisons for FSD were calculated via paired student's t‐test, whereas for GLSS and FWS, Wilcoxon signed rank tests were performed. Correlation analyses were conducted via Spearman correlations. Statistical significance was set at *p* ≤ 0.05. All analyses were conducted using the open source Jamovi software (The Jamovi Project, version 2.3.28, Sydney, Australia).

## 3 Results

### 3.1 Demographics

This study included 18 participants (4 male, 14 female) of Brazilian multi‐ethnic background, with a mean age of 38.6 (7.2) years and a mean BMI of 27.4 (4.7) kg/m^2^ at baseline. The median GLSS at baseline was 3.0 (1.3), whereas the median FWS was 3.0 (1.0), and the mean FSD was 37.17 (27.6) mm (Table 1).

**TABLE 1:** Demographic description of the study sample investigated.

Demographics	Value
Participants	18
Sex	4 males/14 females
Age	38.6 (7.2)
BMI	27.4 (4.7)
Glabella Severity Scale (GLSS)	3.0 (1.3)
Frontal Wrinkle Scale (FWS)	3.00 (1.00)
Frontal Skin Displacement (FSD)	37.17 (27.6)

*Note:* Results are presented as frequency count for participants, as mean and 1× standard deviation for age and BMI, and as median and the respective interquartile range (IQR) for the Glabella Severity Scale (GLSS), Frontal Wrinkle Scale (FWS), and frontal skin displacement (FSD).

### 3.2 Glabella Severity Scale (GLSS)

**FIGURE 4:**
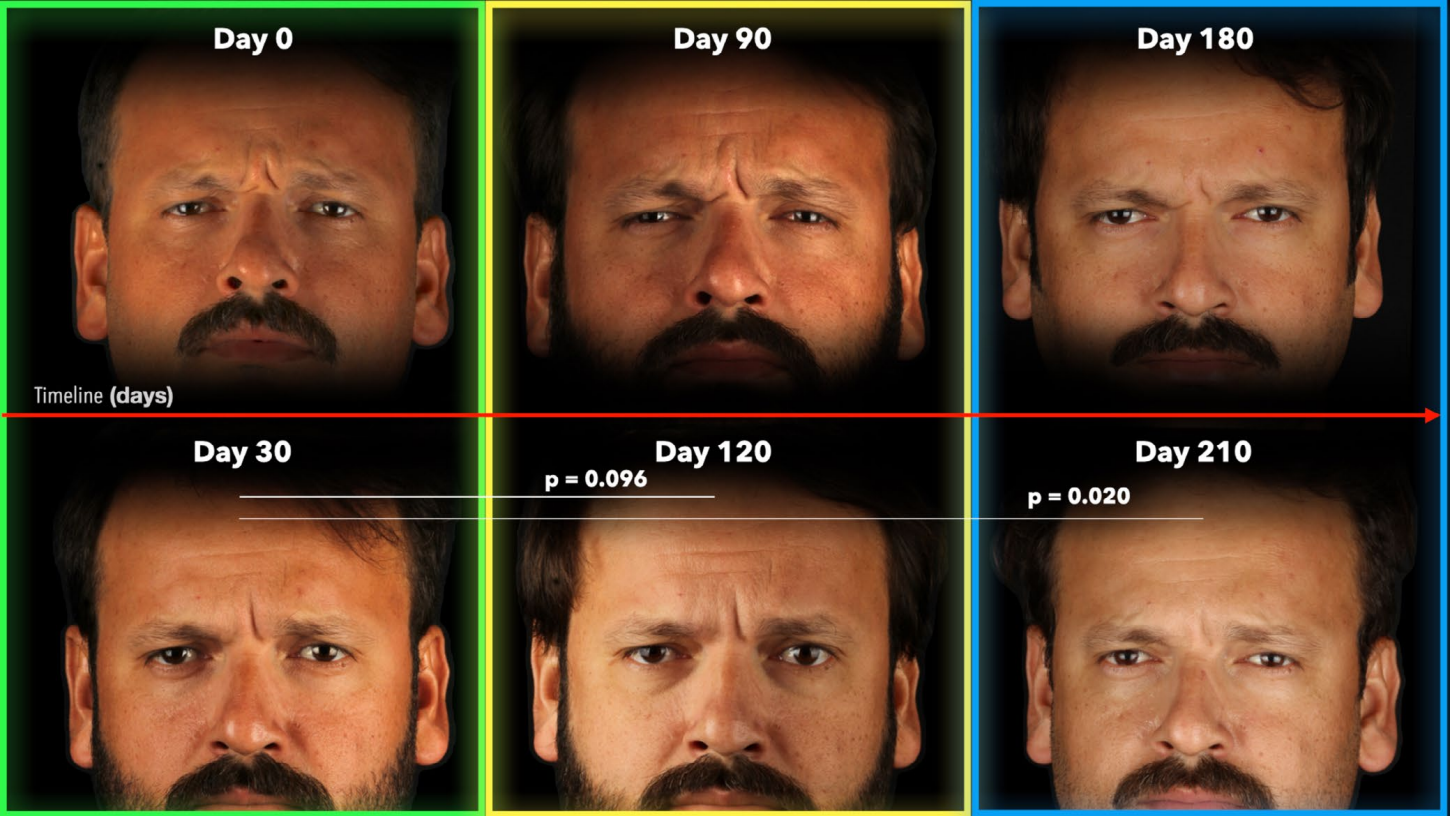
Sequential images of a 42‐year‐old male participant during maximal glabellar contraction. The figure illustrates the progression of wrinkle severity as evaluated by the Glabellar Severity Scale (GLSS) at all treatment (Day 0, 90, and 180) and post‐treatment (Day 30, 120, and 210) endpoints. *p*‐values indicate differences across post‐treatment groups according to the Wilcoxon signed rank test.

**FIGURE 5:**
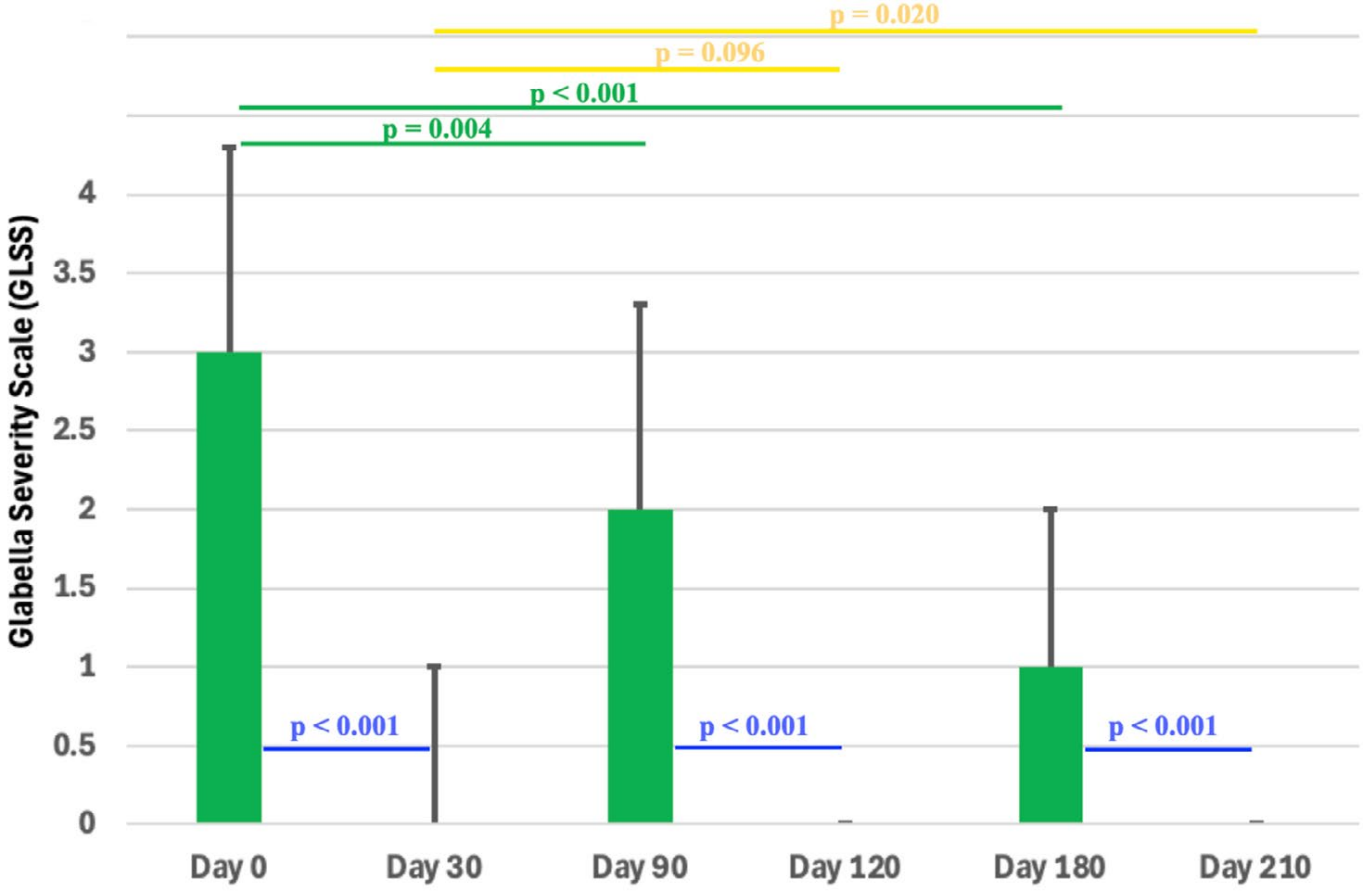
Bar graphs showing the median and interquartile range (IQR) of the Glabella Severity Scale (GLSS) at all treatment (Day 0, 90, and 180) and post‐treatment (Day 30, 120, and 210) endpoints. *p*‐values indicate across‐group differences according to the Kruskal–Wallis test.

Following the first treatment cycle (0 days to 30 days), the GLSS reduced from 3.0 (1.3) to 0.0 (1.0) with *p* < 0.001, whereas in the second treatment cycle (90 to 120 days), the GLSS reduced from 2.0 (1.3) to 0.0 (0.0) with *p* < 0.001, and in the third treatment cycle (180 to 210 days) the GLSS reduced from 1.0 (1.0) to 0.0 (0.0) with *p* < 0.001, respectively. (Figures 4 and 5).

When comparing the GLSS before each treatment cycle (0, 90, 180 days) to the baseline values (= before the beginning of treatment), it was revealed that there was a continuous statistically significant reduction with *p* = 0.004 at 90 days and with *p* < 0.001 at 180 days when compared to baseline (Table 2).

**TABLE 2:** Results showing median and interquartile range (IQR) of the Glabella Severity Scale (GLSS), Frontal Wrinkle Scale (FWS), and eyebrow position scoring (EPS) and mean with 1× standard deviation (SD) of the frontal skin displacement (FSD). Assessments were made at Day 0, 30, 90, 120, 180, and 210 for FWS and FSD, and at Day 30, 120, and 210 for EPS.

Assessment/Time (days)	GLSS median (IQR)	FWS median (IQR)	FSD mean (SD)	EPS median (IQR)
0	3.0 (1.3)	3.0 (1.0)	37.2 (27.6)	
30	0.0 (1.0)	2.0 (2.0)	30.4 (28.6)	0.0 (0.3)
90	2.0 (1.3)	3.0 (1.0)	38.9 (29.1)	
120	0.0 (0.0)	1.0 (1.0)	26.5 (23.8)	0.0 (1.0)
180	1.0 (1.0)	2.0 (2.0)	26.8 (20.1)	
210	0.0 (0.0)	1.0 (1.3)	17.9 (15.0)	0.0 (1.0)

When comparing the GLSS after each treatment cycle (30, 120, 210 days) to the first post‐treatment value at 30 days, it was revealed that there was a continuous reduction with *p* = 0.096 at 120 days and with *p* = 0.020 at 210 days (Table 2; Figure 5).

### 3.3 Forehead Wrinkle Scale (FWS)

**FIGURE 6:**
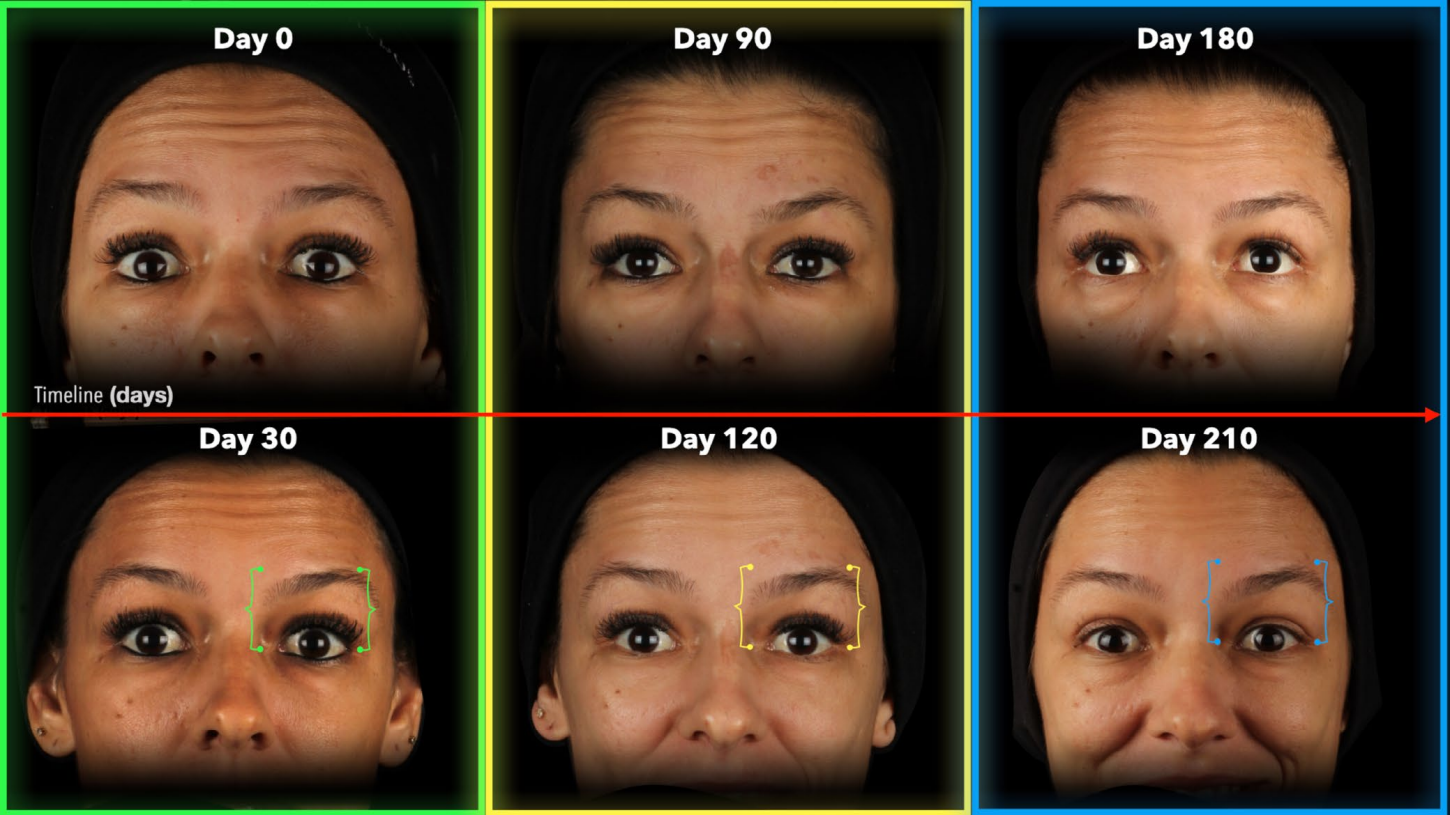
Sequential images of a 37‐year‐old female participant during maximal eyebrow elevation. The figure illustrates changes in forehead lines at all treatment (Day 0, 90, and 180) and post‐treatment (Day 30, 120, and 210) endpoints. Markers aligned with the eyebrow indicate variations in eyebrow elevation across time points.

**FIGURE 7:**
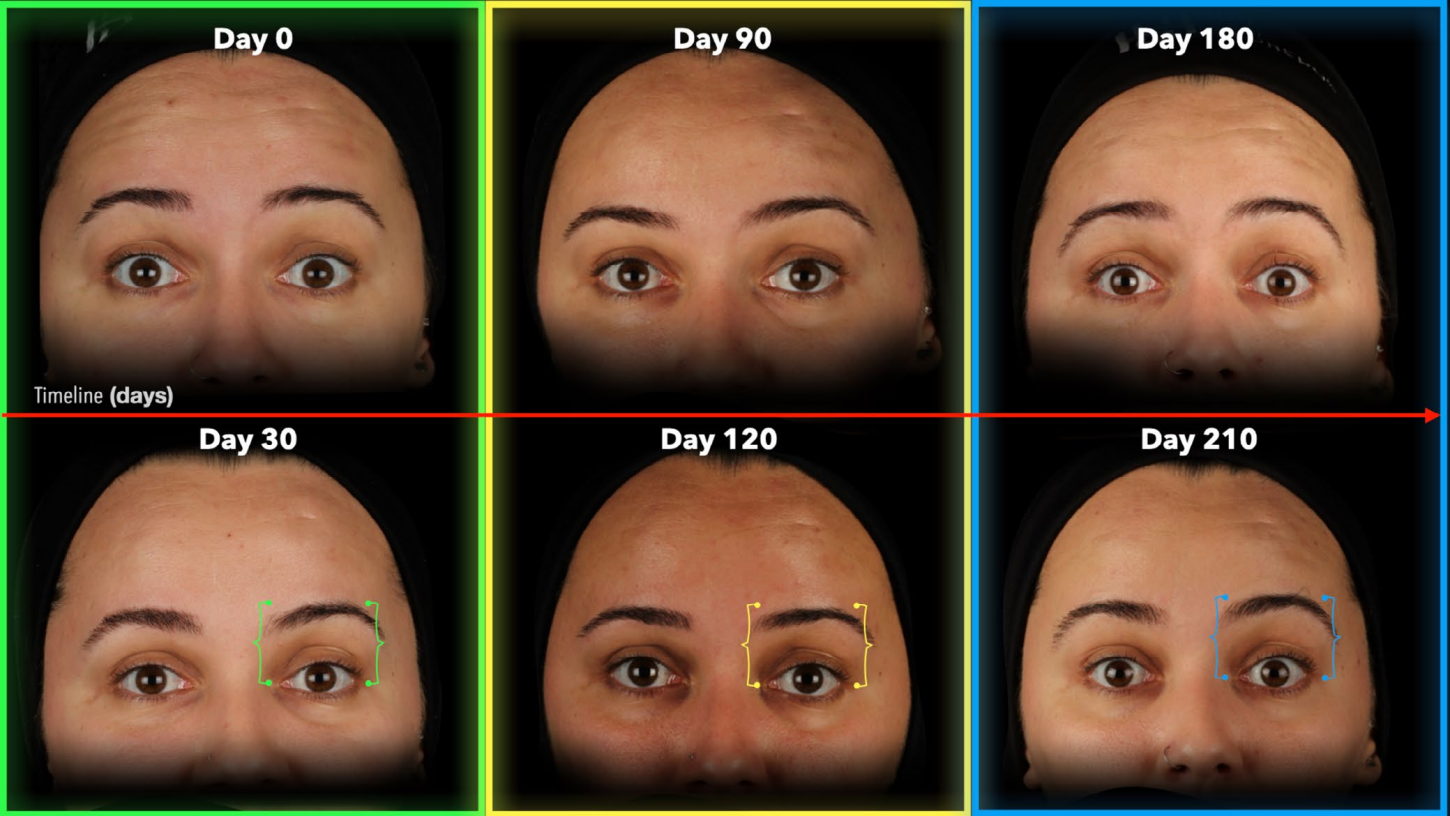
Sequential images of a 35‐year‐old female participant during maximal eyebrow elevation. The figure illustrates changes in forehead lines at all treatment (Day 0, 90, and 180) and post‐treatment (Day 30, 120, and 210) endpoints. Markers aligned with the eyebrow indicate variations in eyebrow elevation across time points.

**FIGURE 8:**
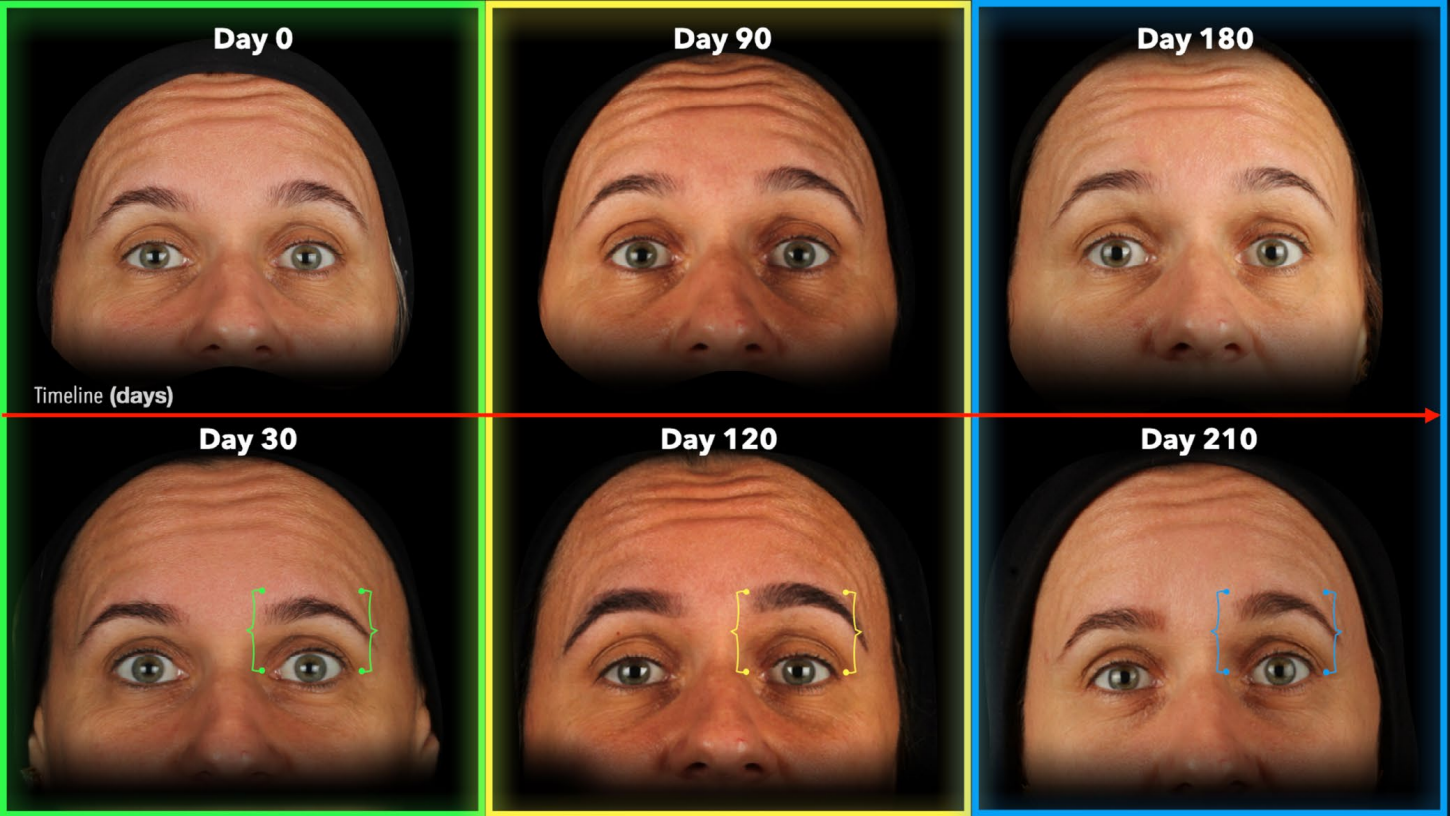
Sequential images of a 46‐year‐old female participant during maximal eyebrow elevation. The figure illustrates changes in forehead lines at all treatment (Day 0, 90, and 180) and post‐treatment (Day 30, 120, and 210) endpoints. Markers aligned with the eyebrow indicate variations in eyebrow elevation across time points.

**FIGURE 9:**
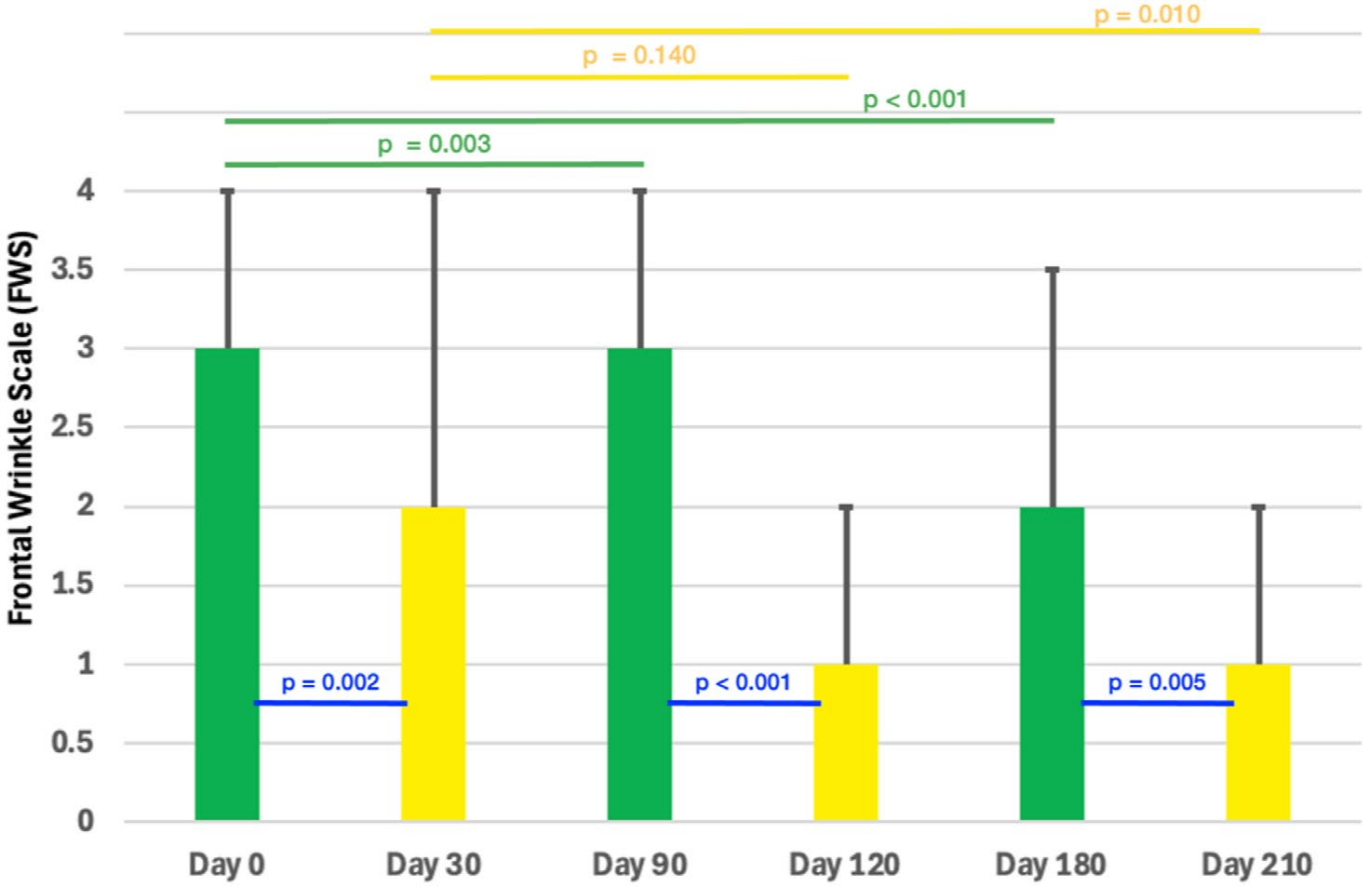
Bar graphs showing the median and interquartile range (IQR) of the Frontal Wrinkle Scale (FWS) at all treatment (Day 0, 90, and 180) and post‐treatment (Day 30, 120, and 210) endpoints. *p*‐values indicate across‐group differences according to Kruskal–Wallis test.

Following the first treatment cycle (0 to 30 days), the FWS reduced from 3.0 (1.0) to 2.0 (2.0) with *p* = 0.002, whereas in the second treatment cycle (90 to 120 days), the FWS reduced from 3.0 (1.0) to 1.0 (1.0) with *p* < 0.001, and in the third treatment cycle (180 to 210 days) the FWS reduced from 2.0 (2.0) to 1.0 (1.3) with *p* = 0.005, respectively (Table 2; Figures 6, 7, 8, 9).

When comparing the FWS before each treatment cycle (0, 90, and 180 days) to the baseline values (= before the beginning of treatment), it was revealed that there was a continuous statistically significant reduction with *p* = 0.003 at 90 days and with *p* < 0.001 at 180 days when compared to baseline.

When comparing the FWS after each treatment cycle (30, 120, and 210 days) to the first post‐treatment value at 30 days, it was revealed that there was a continuous reduction with *p* = 0.140 at 120 days and with *p* = 0.010 at 210 days.

### 3.4 Frontal Skin Displacement (FSD)

**FIGURE 10:**
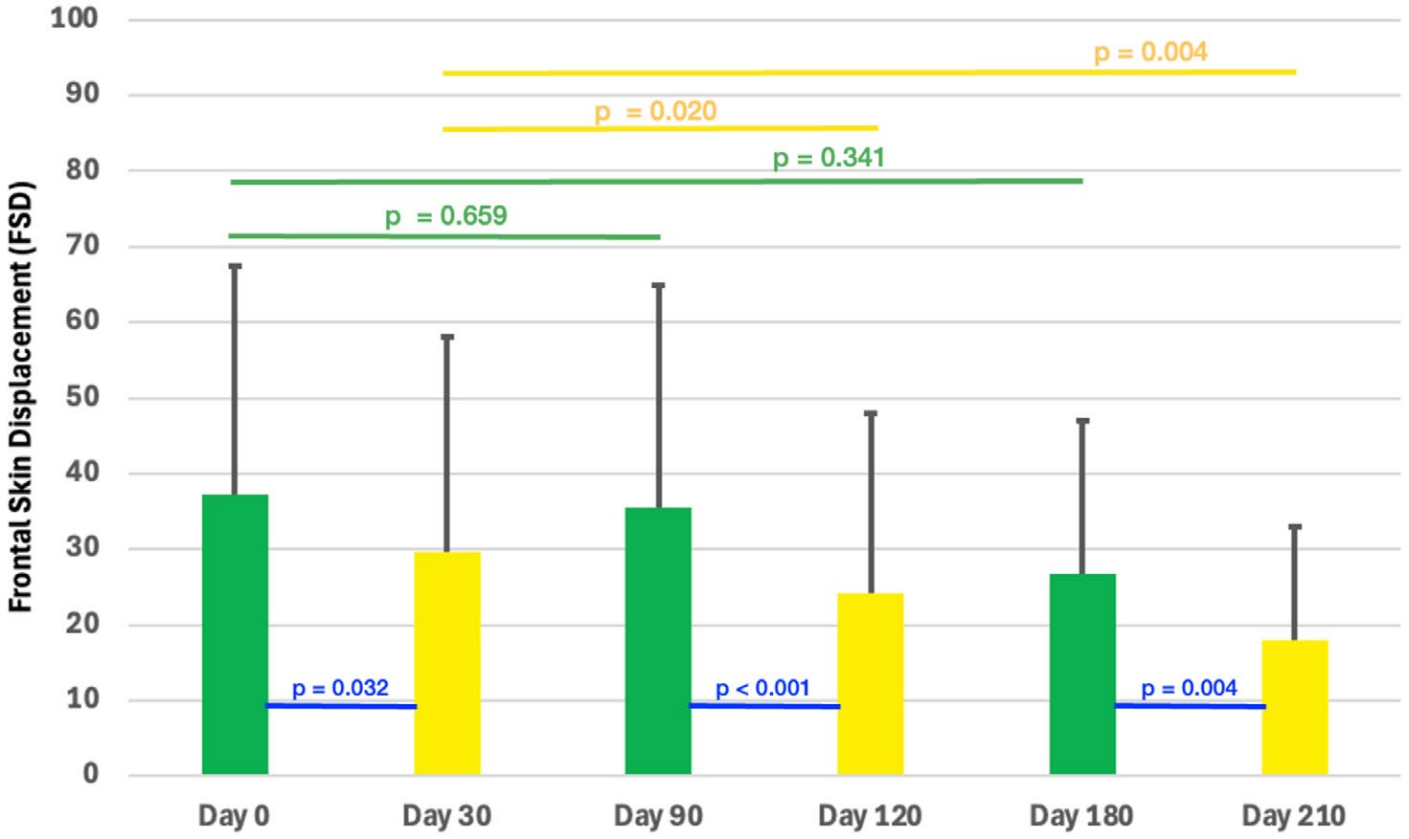
Bar graphs showing the mean and standard deviation (SD) of the Frontal Skin Displacement (FSD) at all treatment (Day 0, 90, and 180) and post‐treatment (Day 30, 120, and 210) endpoints. *p*‐values indicate across‐group differences according to Kruskal–Wallis test.

Pairwise comparisons revealed statistically significant improvements between pre‐ and post‐treatment FSD for each treatment cycle; cycle 1 (0 vs. 30 days): 37.2 (27.6) vs. 30.4 (28.6) mm with *p* = 0.032; cycle 2 (90 vs. 120 days): 38.9 (29.1) vs. 26.5 (23.8) mm with *p* < 0.001; cycle 3 (180 vs. 210 days): 26.8 (20.1) vs. 17.9 (15.0) mm with *p* = 0.004. (Table 2; Figure 10).

Pre‐treatment (0, 90, and 180 days) and post‐treatment (30, 120, and 210 days) comparisons revealed that for the short‐term follow‐up (1 cycle) no statistical significance was detected with *p* = 0.659 and with *p* = 0.341, whereas for the long‐term follow‐up, a statistically significant reduction was observed with *p* = 0.020 and *p* = 0.004 at 180 and 210 days follow‐up, respectively.

### 3.5 Eyebrow Position Scoring (EPS)

The change in eyebrow position after the first treatment cycle was 0.0 (0.3), whereas the change after the second treatment cycle was 0.0 (1.0) and after the third treatment cycle it was 0.0 (1.0), respectively. These results indicate most likely that the treatment did not influence eyebrow position, suggesting that neuromodulator effects remained localized in the glabella without product diffusion to the frontalis muscle across the eyebrow level (Table 2).

### 3.6 Correlation Analyses

When correlating the severity of glabellar lines to the severity of forehead lines, it was discovered that at no timepoint a statistically significant correlation was identified with all *p* > 0.05. This indicates that the FWS is independent of the GLSS, and stronger glabellar contractions are not relatable to more improvement of horizontal forehead lines.

### 3.7 Adverse Events

No adverse events were observed during the study observational period (210 days) that could be related to the neuromodulator treatment, like medial eyebrow ptosis, upper eyelid ptosis, or lateral hyper‐elevation (Spock eyebrow). No follow‐up injections were requested by the patients or seemed necessary by the initial treatment provider.

## 4 Discussion

This prospective clinical interventional pilot study was designed to identify the effects of glabellar injections on the severity of frontal rhytids. The results revealed that even though the forehead was not addressed with neuromodulators, the severity of frontal rhytids improved on a statistically significant level. Moreover, conducting a total of three treatment cycles with each having an observational period of at least 3 months, it was identified that continuous glabellar treatments reduced the severity of frontal rhytids on a statistically significant basis.

The interplay between facial elevators and facial depressors when using neuromodulator treatments has long been known to the injector community and was summarized under the term: *facial biomechanics* [13]. Despite being introduced already in 1985 to the scientific literature, this term became more established in the context of facial aesthetics when the interplay between different anatomic structures was described [13]. The modern understanding of the term facial biomechanics in the context of facial muscle function describes the complex interplay between various facial muscles that interact together and form muscle complexes that ultimately move the skin in various directions [12]. The knowledge about individual and combined functional muscle anatomy is crucial to identify the one or the group of muscles that are responsible for a specific aesthetic clinical presentation, especially if a treatment with neuromodulators is planned. Elevating the tail of the eyebrow, for instance, is possible [14] if the knowledge is available that the frontalis muscle acts as an eyebrow elevator, whereas the lateral vertical fibers of the orbicularis oculi muscle act as a lateral eyebrow depressor [15, 16]. Therefore, placing small doses of neuromodulators below the tail of the eyebrow can result clinically in lateral eyebrow elevation. However, this is only possible if the frontalis muscle is capable of exerting sufficient elevator function to increase the height of the tail; targeting the depressor muscle without sufficient elevator function would have a limited clinical effect.

The procerus and corrugator supercilii muscles act as medial and central eyebrow depressors and oppose the elevator function of the frontalis muscle, which is the sole elevator of the medial, central, and lateral thirds of the eyebrow [17]. A recent MRI‐based study by Rams et al. [5] described that during various facial expressions like repose, anger, joy, surprise, and sadness, the respective antagonistic pairs are both active at the same time. This finding is novel because it shows that both antagonistic pairs (depressor and elevator muscles) tense up simultaneously and are not only active when their individual functions are needed: the frontalis muscle increased in thickness during each of the above‐mentioned facial expressions, including the facial expression of anger, which results in eyebrow depression. Understanding that the co‐activation of the antagonistic pairs is needed to precisely and quickly position the eyebrows allows practitioners to estimate desired treatment outcomes following patient assessment.

Our study utilizes the knowledge of facial biomechanics as described above and applies it to upper facial neuromodulator treatments. The results of our investigation revealed that the GLSS continuously improved from 3.0 (before the treatment) to 2.0 at 3 months and to 1.0 at 6 months; this can be regarded as a long‐term effect following repetitive glabellar neuromodulator injections with 37.5 sU (= 15 IU) of the neuromodulator product. FWS and FSD evaluation revealed that each glabellar treatment resulted in a statistically significant improvement in forehead rhytid severity and forehead skin movement with all *p* ≤ 0.05. This clinical outcome is surprising and novel because the forehead was not targeted during the neuromodulator treatments (Figures 5 and 8).

The supportive evaluation of the position of the eyebrow via the conducted EPS analyses revealed that at all follow‐up time points (30, 120, and 210 days) no change in the ability to elevate eyebrows or in the height of the eyebrows under maximal frontalis muscle contraction was detected, with all 0.0; indicating no change to the position of the eyebrows when compared to before the treatments. The utilized product reconstitution and the deployed injection points (3‐point injection technique) were selected specifically to assure that the frontalis muscles remained unaffected by the injected product.

The most likely explanation for the results detected in this study (indirect FWS improvement despite not addressing the forehead) is that due to the reduction in contraction strength of the medial and central eyebrow depressors (= procerus and corrugator supercilii muscles), the opposing eyebrow elevator (= frontalis muscle) had to exert less force to maintain the position or to move the eyebrows. In light of facial biomechanics, this is plausible and follows the basic understanding principles of antagonistic pairs of eyebrow elevators and depressors, which work in conjunction with each other. Reducing the force of the depressor will positively affect the elevator, which clinically results in reduced rhytid severity. The fact that this change did not achieve its maximal strength during the first treatment cycle but only during the second and third cycles supports these biomechanical principles [18, 19, 20]. The reduction in depressor strength resulted in a gradual adaptation of the frontalis muscle's effect when looking at Day 30, 120, 210: FWS: 2.0 vs. 1.0 vs. 1.0 and FSD: 30.4 mm vs. 26.5 vs. 17.9 mm (Table 2).

The observed results suggest a novel approach to preventing forehead wrinkles, differing significantly from current practices: treating the forehead by not injecting the forehead but instead injecting the glabellar muscles with the 3‐point injection technique. This method may also help preserve the elevator function of the frontalis muscle, which is especially beneficial in mature patients with idiopathic eyebrow ptosis. Future studies will need to explore more the findings presented in this pilot study and address some of the limitations inherent in this study: a larger sample size, a longer study follow‐up, a more diverse study population, the use of other neuromodulator products, and potentially including electromyographic confirmation for providing evidence that the frontalis muscle was not affected by the deployed injection algorithm and product reconstitution.

## 5 Conclusion

Our findings support the hypothesis that neuromodulator injections in the glabellar region can indirectly improve forehead rhytids by altering the balance between eyebrow depressors and elevators. Moreover, the observed progressive improvement across treatment cycles suggests that this strategy may continuously enhance aesthetic outcomes over time, supporting the rationale for maintenance treatments at regular intervals, such as every 3 months. By focusing on the glabellar complex, clinicians can achieve significant improvements in forehead line severity by using less product for the frontalis muscle or even without the need for direct frontalis muscle injections, thereby minimizing the risk of adverse events such as eyelid or eyebrow ptosis.

## Author Contributions

All authors significantly contributed to the conception, design, and execution of this study. They participated in data collection, analysis, and interpretation, as well as in drafting and critically revising the manuscript. Each author has reviewed and approved the final version of the article and agrees to be accountable for all aspects of the work, ensuring its integrity and accuracy.

## Conflicts of Interest

The authors declare no conflicts of interest.

## Data Availability

The data that support the findings of this study are available on request from the corresponding author. The data are not publicly available due to privacy or ethical restrictions.

## References

[1] American Society of Plastic Surgeons , “2023 ASPS Procedural Statistics Release,” 2023 1–41. 10.1097/01.prs.0001028284.06979.be 40556463

[2] D. S. Chauhan , K. M. Cariappa , and Y. Guruprasad , “Botulinum Toxin Type A for the Treatment of Hyperkinetic Lines of the Face,” Journal of Maxillofacial and Oral Surgery 12, no. 2 (2013): 173–183, 10.1007/s12663-012-0407-1. 24431836 PMC3681999

[3] A. Carruthers , J. Carruthers , K. De Boulle , N. Lowe , E. Lee , and M. F. Brin , “Treatment of Crow's Feet Lines and Forehead Lines With Botox (onabotulinumtoxinA): Development, Insights, and Impact,” Medicine 102, no. S1 (2023): e32496, 10.1097/MD.0000000000032496. 37499083 PMC10374187

[4] S. Cotofana , N. Solish , C. Gallagher , K. Beleznay , C. A. Hernandez , and V. Bertucci , “The Anatomy Behind Eyebrow Positioning: A Clinical Guide Based on Current Anatomic Concepts,” Plastic and Reconstructive Surgery 149, no. 4 (2022): 869–879, 10.1097/PRS.0000000000008966. 35139063

[5] D. J. Rams , M. Koziej , S. M. Shridharani , et al., “Exploratory Analysis of Upper Facial Muscle Interplay During Emotional Expressions: Magnetic Resonance Imaging (MRI) Insights From Young, Caucasian, Toxin‐Naïve Individuals,” Aesthetic Surgery Journal 45, no. 4 (2025): 414–421, 10.1093/asj/sjae246. 39696998

[6] M. Alfertshofer , N. Engerer , K. Frank , N. Moellhoff , D. L. Freytag , and S. Cotofana , “Multimodal Analyses of the Aging Forehead and Their Clinical Implications,” Aesthetic Surgery Journal 43, no. 7 (2023): NP531–NP540, 10.1093/asj/sjad009. 36647564

[7] T. C. Flynn , A. Carruthers , J. Carruthers , et al., “Validated Assessment Scales for the Upper Face,” Dermatologic Surgery 38, no. 2 (2012): 309–319, 10.1111/j.1524-4725.2011.02248.x. 22316187

[8] S. Cotofana , A. P. Pedraza , J. Kaufman , et al., “Respecting Upper Facial Anatomy for Treating the Glabella With Neuromodulators to Avoid Medial Brow Ptosis—A Refined 3‐Point Injection Technique,” Journal of Cosmetic Dermatology 20, no. 6 (2021): 1625–1633, 10.1111/jocd.14133. 33817912

[9] V. R. M. Munoz‐Lora , V. Thiesen , D. Loureiro , et al., “Understanding Clinical Meaningfulness When Targeting the Depressor Anguli Oris Muscle (DAO) With Neuromodulators: A Clinical Prospective Interventional Study,” Journal of Neural Transmission (2024), 10.1007/s00702-024-02835-6. 39276240

[10] M. Germani , C. C. M. S. Almeida , V. R. M. Munoz‐Lora , et al., “How to Improve Infraorbital Hollows With Neuromodulators–A Clinical Prospective Interventional Study About the Application of Facial Biomechanics,” Journal of Cosmetic Dermatology 22, no. 11 (2023): 2950–2956, 10.1111/jocd.15970. 37632259

[11] A. Carruthers , J. Carruthers , B. Hardas , et al., “A Validated Grading Scale for Forehead Lines,” Dermatologic Surgery 34 (2008): S155–S160, 10.1111/j.1524-4725.2008.34364.x. 19021673

[12] V. Rogerio , J. B. Carvas , M. G. Vieira , V. Rabelo , P. Roschel , and V. R. M. Munoz‐Lora , “3D Stereophotogrammetry Quantification for Tissue Repositioning Using Botulinum Toxin A: A Case Report,” Brazilian Dental Science 25, no. 3 (2022): e3411, 10.4322/bds.2022.e3411.

[13] L. Freytag , M. G. Alfertshofer , K. Frank , et al., “Understanding Facial Aging Through Facial Biomechanics,” Facial Plastic Surgery Clinics of North America 30, no. 2 (2022): 125–133, 10.1016/j.fsc.2022.01.001. 35501049

[14] S. Jabbour , C. Awaida , E. Kechichian , et al., “Botulinum Toxin for Eyebrow Shaping: A Systematic Review,” Dermatologic Surgery 43, no. 3 (2017): S252–S261, 10.1097/DSS.0000000000001410. 33065951

[15] C. Angrigiani , F. Felice , A. O. Rancati , et al., “The Anatomy of the Frontalis Muscle Revisited: A Detailed Anatomic, Clinical, and Physiologic Study,” Aesthetic Surgery Journal 44, no. 6 (2024): 565–571, 10.1093/asj/sjad320. 37768166

[16] S. Cotofana , D. L. Freytag , K. Frank , et al., “The Bidirectional Movement of the Frontalis Muscle: Introducing the Line of Convergence and Its Potential Clinical Relevance,” Plastic and Reconstructive Surgery 145, no. 5 (2020): 1155–1162, 10.1097/PRS.0000000000006756. 32332530

[17] A. C. Abramo , T. P. A. Do Amaral , B. P. Lessio , and G. A. De Lima , “Anatomy of Forehead, Glabellar, Nasal and Orbital Muscles, and Their Correlation With Distinctive Patterns of Skin Lines on the Upper Third of the Face: Reviewing Concepts,” Aesthetic Plastic Surgery 40, no. 6 (2016): 962–971, 10.1007/s00266-016-0712-z. 27743084

[18] A. D. Nassif , R. F. Boggio , S. Espicalsky , and G. E. L. Faria , “High Precision Use of Botulinum Toxin Type A (BONT‐A) in Aesthetics Based on Muscle Atrophy, Is Muscular Architecture Reprogramming a Possibility? A Systematic Review of Literature on Muscle Atrophy After BoNT‐A Injections,” Toxins 14, no. 2 (2022): 81, 10.3390/toxins14020081. 35202109 PMC8878196

[19] F. Ingallina , K. Frank , S. Mardini , et al., “Reevaluation of the Layered Anatomy of the Forehead: Introducing the Subfrontalis Fascia and the Retrofrontalis Fat Compartments,” Plastic and Reconstructive Surgery 149, no. 3 (2022): 587–595, 10.1097/PRS.0000000000008826. 35006205

[20] F. Ingallina , M. G. Alfertshofer , L. Schelke , et al., “The Fascias of the Forehead and Temple Aligned—An Anatomic Narrative Review,” Facial Plastic Surgery Clinics of North America 30, no. 2 (2022): 215–224, 10.1016/j.fsc.2022.01.006. 35501059

